# A Clinical-Radiomics Nomogram Based on Magnetic Resonance Imaging for Predicting Progression-Free Survival After Induction Chemotherapy in Nasopharyngeal Carcinoma

**DOI:** 10.3389/fonc.2022.792535

**Published:** 2022-06-22

**Authors:** Lu Liu, Wei Pei, Hai Liao, Qiang Wang, Donglian Gu, Lijuan Liu, Danke Su, Guanqiao Jin

**Affiliations:** ^1^ Department of Radiology, Guangxi Medical University Cancer Hospital, Nanning, China; ^2^ Department of Anesthesiology, Guangxi Medical University Cancer Hospital, Nanning, China

**Keywords:** nasopharyngeal carcinoma, radiomics, magnetic resonance imaging, induction chemotherapy, survival models

## Abstract

**Purpose:**

This paper aimed to establish and verify a radiomics model based on magnetic resonance imaging (MRI) for predicting the progression-free survival of nasopharyngeal carcinoma (NPC) after induction chemotherapy (IC).

**Materials and Methods:**

This cohort consists of 288 patients with clinical pathologically confirmed NPC, which was collected from January 2015 to December 2018. All NPC patients were randomly divided into two cohorts: training (n=202) and validation (n=86). Radiomics features from the MRI images of NPC patients were extracted and selected before IC. The patients were classified into high- and low-risk groups according to the median of Radscores. The significant imaging features and clinical variables in the univariate analysis were constructed for progression-free survival (PFS) using the multivariate Cox regression model. A survival analysis was performed using Kaplan–Meier with log-rank test and then each model’s stratification ability was evaluated.

**Results:**

Epstein–Barr virus (EBV) DNA before treatment was an independent predictor for PFS (p < 0.05). Based on the pyradiomic platform, we extracted 1,316 texture parameters in total. Finally, 16 texture features were used to build the model. The clinical radiomics-based model had good prediction capability for PFS, with a C-index of 0.827. The survival curve revealed that the PFS of the high-risk group was poorer than that of the low-risk group.

**Conclusion:**

This research presents a nomogram that merges the radiomics signature and the clinical feature of the plasma EBV DNA load, which may improve the ability of preoperative prediction of progression-free survival and facilitate individualization of treatment in NPC patients before IC.

## Introduction

Nasopharyngeal carcinoma (NPC) is a malignant tumor related to Epstein–Barr virus (EBV) infection that arises from the mucosal epithelium of the nasopharynx, and it has a predilection for the lateral and posterior apical walls of the nasopharyngeal cavity (1, 2). It has distinct geographical distribution characteristics, especially in East Asia and Southeast Asia (3, 4). According to the global tumor epidemiology statistics of the International Agency for Research on Cancer, in 2020, it was estimated that there would be 133,000 new cases of nasopharyngeal cancer and 80,000 deaths worldwide, about half of them from China, especially in the Guangdong and Guangxi provinces.

Currently, concurrent chemoradiotherapy (CCRT) is the standard-of-care treatment for patients with locally advanced NPC, as chemotherapy sensitizes tumor cells to the toxic effect of radiotherapy. Combination induction or neoadjuvant chemotherapy and chemoradiotherapy has been investigated in these patients (5, 6). Recently, a number of clinical trials have shown that IC + CCRT regimen shows significantly better survival benefit compared with CCRT alone (7–10). Thus, the clinical treatment decisions of the NCCN Guidelines (2020.V2) have also been changed; IC + CCRT regimen was recommended as category 2A.

However, physicians need to identify patients who are at high risk of early progression and tailor the treatment strategies. At present, clinical therapeutic regimens for NPC patients are mainly based on the tumor node metastasis (TNM) staging system, which is related to the anatomy-only basis. However, this staging system is far from sufficient to predict the survival benefit of therapeutic strategies and cannot reflect the likelihood of cancer invasion and metastasis. Furthermore, patients with the same TNM stages, who usually received the same therapeutic regimens, will have different outcomes. Recognizing early those patients who can benefit from intensified induction chemotherapy (IC) would allow the modification of treatment regimens in time (11–16), which is important for maximizing efficacy, minimizing therapeutic toxicities, and serving the purpose of precision oncology. We urgently need a better approach to identify NPC patients who are best suitable for additional IC (17).

Radiomics, which is being evaluated as a popular data-mining method, extracts features from medical imaging in a high-throughput and automated way to reflect its subtle heterogeneous phenotypes for various cancers (18). Recently, radiomics has been applied to patients with head and neck cancer, and it is reported to improve prognostic performance versus the TNM staging system (19, 20). Recent studies showed that the texture parameter of pretreatment magnetic resonance imaging (MRI) or MRI-based radiomics nomogram could predict progression-free survival (PFS) in endemic NPC (21). Peng et al. established a PET/CT radiomics model to divide NPC patients into a low-risk group and a high-risk group, and there were significant differences in the 5-year disease-free interval (DFI) between the groups. All the characteristics were significantly correlated with the DFI rate, which was better than the plasma EBV DNA-based model for risk stratification and selection-guided induction chemotherapy (IC) regimen (22). Zhao et al. confirmed that MRI texture features also have good performance in evaluating the efficacy of IC. Risk stratification of patients with the help of radiomics models before treatment and accurate assessment of efficacy after treatment are helpful to formulate and adjust individualized treatment strategies to avoid unnecessary adverse reactions and medical expenses (23).

Herein, our study aimed to establish a radiomics-based model based on MRI for predicting the PFS of NPC after IC. We hypothesized that this model could stratify the NPC patients into high-risk and low-risk groups before IC. It will help physicians tailor the treatment strategies and choose the most appropriate treatment.

## Materials and Methods

### Patients

This retrospective research was approved by the Institutional Review Board of our institution, and informed consent was waived from all patients. In total, 288 patients with clinical pathologically confirmed NPC without evidence of locoregional recurrence or distant metastases at diagnosis were randomly recruited into 2 cohorts in a 7:3 ratio: training (n = 202; mean age, 44.69 ± 11.02 years) and validation (n = 86; mean age, 43.93 ± 11.03 years), which were collected from January 2015 to December 2018 in Guangxi Medical University Cancer Hospital. The following inclusion criteria were met: (1) pathologically diagnosed NPC; (2) images of contrast-enhanced MRI before treatment; and (3) treatment modalities, including upfront IC, and subsequent radiotherapy or CCRT. The exclusion criteria were as follows:(1) patients without contrast-enhanced MRI before induction chemotherapy, or the data of the Digital Imaging and Communications in Medicine (DICOM) were corrupted and images had unclear lesion boundaries or artifacts; (2) patients with incomplete clinical data; and (3) patients with distant metastasis before treatment.

### MRI Scan

All patients underwent 3.0T MRI (Discovery MR750; GE Healthcare, Little Chalfont, UK) scans before treatment using a standard head–neck combined coil. The scanned region extended from the upper edge of the suprasellar cistern to the inferior edge of the sternal end of the clavicle. T1-weighted turbo spin-echo (TSE) sagittal, axial, and coronal sequences were as follows: repetition time (TR)=957 ms; echo time (TE)=19 ms; field of view (FOV)=240×240 mm; slice thickness=5.0 mm; slice gap=1.0 mm; frequency matrix=256×256; T2-weighted fat-suppressed axial sequences were as follows: TR=6,760 ms; TE=91 ms; FOV= 240×240 mm; slice thickness=5.0 mm; slice gap=1.0 mm; frequency matrix=384×384, following an intravenous bolus of 0.2 ml/kg Gadopentetate Dimeglumine (Magnevist, Schering, Berlin, Germany). The T1-weighted fat-suppressed sagittal, axial, and coronal sequences were performed using the same parameters as those of the T1-weighted before the injection.

### Radiomics Workflow

The radiomics workflow consists of five steps is shown in **Supplementary Figure 1**, including acquisition of image, image segmentation, extracted features, feature selection, and modeling.

## Follow-up and Clinical Endpoint

Patients were reexamined every three months for the first post-treatment year, then every six months next year, and yearly thereafter by CT or MRI examinations routinely, while nasopharyngoscopy examinations were performed when necessary. PFS was used as the survival endpoint, which reflected the efficacy and required a relatively short follow-up. PFS was calculated from the date of the initial treatment to the date of first relapse (local or distant metastases), death, or the last follow-up (censored).

### Image Acquisition, Image Segmentation, and Feature Extraction

We obtained MRI images (axial CE-T1-weighted and axial fat-saturated T2-weighted imaging series) from the Picture archive communication systems (PACS) for extracting radiomics features. MRI images were exported to the ITK-Snap software (open-source software; www.itk-snap.org) for manual three-dimensional segmentation. All manual segmentations of the tumor were conducted by two senior radiologists (who had 15 and 20 years of experience in NPC imaging, respectively); the intraclass correlation coefficient (ICC) was calculated to assess interobserver agreement, with ICC > 0.75 regarded as “excellent” (ICC was 0.912). The region of interest (ROI) was drawn manually to include the entire tumor and was delineated on each layer of both the axial T2-w images and CET1-w images. Subsequently, numerous texture parameters were extracted based on the pyradiomic platform (version 2.1.2; https://pyradiomics.readthedocs.io/en/latest/index.html).

### Feature Selection, Model Construction, and Validation

Feature selection and model construction of NPC patients were performed in the training set, while the validation set was used for model validation. The least absolute shrinkage and selection operator (LASSO) method was utilized to select the optimal features and avoid overfitting. The coefficients of the less contributive variables were compressed to zero by penalty terms, and the remaining features of nonzero coefficients related to survival were evaluated with the univariate Cox analyses. The Radscore for each NPC patient was obtained by summing the weighted significant features together (Radscore = coefficient 1× feature 1 + coefficient 2× feature 2…) through a linear combination (24). Every patient was classified into a high- or low-risk group subsequently based on the median of the Radscore. The clinical model and radiomic model were established using the Cox proportional hazards model. We combined the radiomics with pretreatment clinical characteristics to construct a radiomics–clinical model with multivariate Cox analyses. Survival analysis was performed using Kaplan–Meier with log-rank test and then each model’s stratification ability was evaluated. To assess the model predictive ability, the concordance index (C-index) was calculated. The prognostic nomogram of PFS was constructed based on the highest C-index model to display individual contributions. The actual scores correspond to the above point coordinate. Calibration curves were plotted to show how closely the model’s prediction agree with the actual result.

### Statistical Analysis

The nominal variable was compared with chi-square test or Fisher’s exact test, while the ordinal variable was evaluated by Kruskal-Wallis H-test. An abnormally distributed continuous variable was evaluated using Mann-Whitney test. All statistical analyses for this study were carried out with R (version 3.5.1) and Python (version 3.5.6). The detailed R packages are given in **Supplementary Methods 1**. A two-tailed *p*-value < 0.05 indicated statistical significance. We used C-index, AUC, ACC, Sensitivity, and Specificity to quantify the discrimination ability. DeLong test was used to compare the AUCs. Decision curve analysis was conducted to estimate the models’ clinical usefulness. Significant clinical variables were selected by the univariate Cox regression analysis, and then we built a clinical radiomics model with multivariate Cox regression analysis.

## Results

### General Demographic and Clinical Characteristics

A total of 288 patients were enrolled in our study. The clinical characteristics of the patients are listed in **Table 1**. The difference in terms of the baseline clinical characteristics (age, gender, TNM stage, or EBV DNA) was not statistically significant between the training and validation cohorts (*p*>0.05). Among the 288 enrolled patients, 98% received radiotherapy.

**Table 1:** Characteristics of the patients in the training and validation cohorts.

Variable	Training (N = 202)	Validation (N = 86)	*p*-value
EBV.DNA0	128 (63.37%)	57 (66.28%)	0.637
EBV.DNA1	74 (36.63%)	29 (33.72%)	
Male	152 (75.25%)	68 (79.07%)	0.485
Female	50 (24.75%)	18 (20.93%)	
Family history	35 (17.33%)	17 (19.77%)	0.962
No family history	167 (82.67%)	69 (80.23%)	
Smoking	70 (34.65%)	31 (36.05%)	0.61
No smoking	132 (74.25%)	46 (63.95%)	
T_stage1	4 (1.98%)	2 (2.33%)	0.641
T_stage2	59 (29.21%)	24 (27.91%)	
T_stage3	53 (26.24%)	28 (32.56%)	
T_stage4	86 (42.57%)	32 (37.21%)	
N_stage0	4 (1.98%)	0 (0.00%)	0.199
N_stage1	53 (26.24%)	31 (36.05%)	
N_stage2	81 (40.10%)	33 (38.37%)	
N_stage3	64 (31.68%)	22 (25.58%)	
Clin_stage3	66 (32.68%)	36 (41.86%)	0.137
Clin_stage4	136 (67.33%)	50 (58.14%)	
WHO2	18 (8.91%)	9 (10.47%)	0.53
WHO3	182 (90.10%)	77 (89.53%)	
WHO4	2 (0.99%)	0 (0.00%)	
Height	1.65 (1.58, 1.70)	1.66 (1.60, 1.70)	0.684
Weight	59.00 (52.00, 67.03)	60.00 (54.00, 67.00)	0.709
Dosage	72.32 (71.66, 72.60)	72.32 (70.40, 72.60)	0.576
Radiotherapy course	32.00 (32.00, 33.00)	32.00 (32.00, 33.00)	0.726
Leukocyte	7.09 (5.88, 8.48)	6.95 (5.81, 8.24)	0.793
Hemoglobin	137.00 (125.95, 148.00)	140.00 (126.90, 149.15)	0.467
Platelets	275.50 (232.00, 325.05)	291.00 (237.90, 332.00)	0.361
Neutrophil	4.50 (3.40, 5.46)	4.23 (3.29, 5.28)	0.549
Lymphocytes	1.72 (1.36, 2.23)	1.81 (1.48, 2.31)	0.211
Albumin	39.80 (37.60, 41.70)	39.55 (37.50, 41.91)	0.808
LDH	173.50 (151.95, 203.15)	183.50 (152.95, 210.10)	0.229
Age	44.69 ± 11.02	43.93 ± 11.03	0.592
PFS (months)	25.58	43.24	0.000

### Clinical Characteristics

EBV DNA before treatment was an independent predictor for PFS (*p<*0.05). The C-index based on the clinical nomogram was 0.716 (95% CI, 0.573–0.860) in the training cohort and 0.603 (95% CI, 0.370–0.836) in the validation cohort. In the training cohort, the AUC, ACC, Sensitivity, and Specificity were 0.583, 55.40%, 43.30%, and 73.20%, respectively, and in the validation cohort, they were 0.655, 67.4%, 51.4%, and 79.6%, respectively (**Table 3**).

### Performance of Model in Predicting PFS

**Figure 1:**
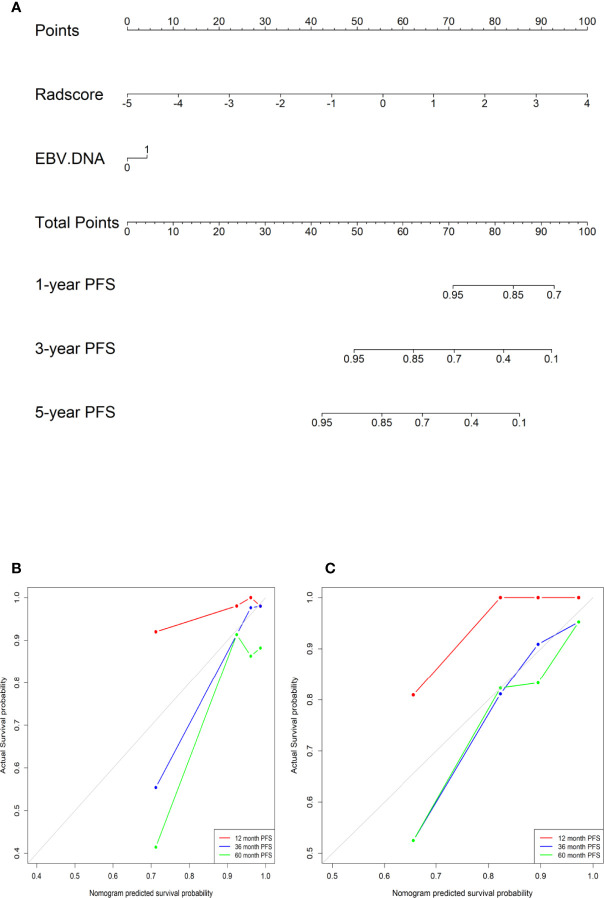
**(A)** A radiomic nomogram integrating the radiomic signature and EBV DNA. **(B)** The calibration curves in the training cohort. **(C)** The calibration curves in the validation cohort.

A total of 1,316 radiomic features were extracted from VOI on axial T2-w and CET1-w images, respectively. The final 16 remaining features were used to build the model (**Figure 1A**). We calculated the Radscore reflecting the risk of PFS of each patient using the formula above. The optimal cutoff value is the median Radscore (-0.229), which was used to divide the patients into high- and low-risk groups. At last, 16 radiomic features were selected to build the nomogram (**Table 2**). The C-Index, AUC, ACC, Sensitivity, and Specificity of the radiomics model (only based on the imaging features) in the training cohort were 0.818, 0.734, 69.30%, 63.30%, and 78.00%, respectively, and in the validation cohort, they were 0.746, 0.606, 64.00%, 51.40%, and 73.50%, respectively (**Table 3**). The predictive value of PFS was increased in the clinical radiomics model [incorporating both clinical variables and imaging features, in both the training cohort (*p* = 0.039, Delong test) and the validation cohort (*p* = 0.015, Delong test)]. The C-index, AUC, ACC, Sensitivity, and Specificity of the clinical radiomics model (only based on the imaging features) in the training cohort were 0.827, 0.777, 74.80%, 82.50%, and 63.40%, respectively, and in the validation cohort, they were 0.751, 0.695, 69.80%, 62.20%, and 75.50%, respectively (**Table 3**).

**Table 2:** Radiomic feature selection result.

MRI series	Selected features (CET1-w + T2-w)
CET1-w	T1c_wavelet.HHL_glszm_SmallAreaLowGrayLevelEmphasis
CET1-w	T1c_logarithm_firstorder_Skewness
CET1-w	T1c_wavelet.HHL_glrlm_ShortRunLowGrayLevelEmphasis
CET1-w	T1c_wavelet.HLL_glcm_Correlation
CET1-w	T1c_wavelet.LLL_glcm_MCC
CET1-w	T1c_wavelet.HHL_firstorder_Median
CET1-w	T1c_wavelet.LHH_gldm_DependenceVariance
CET1-w	T1c_wavelet.LHL_gldm_SmallDependenceLowGrayLevelEmphasis
CET1-w	T1c_wavelet.LHH_glcm_InverseVariance
T2-w	T2_logarithm_glcm_ClusterProminence
T2-w	T2_wavelet.HLH_firstorder_Median
T2-w	T2_wavelet.HLH_firstorder_Mean
T2-w	T2_wavelet.HHH_glcm_InverseVariance
T2-w	T2_exponential_glszm_GrayLevelVariance
T2-w	T2_wavelet.LLH_firstorder_Median
T2-w	T2_original_shape_MajorAxisLength
–	EBV.DNA

CET1-w, contrast-enhanced T1-weighted; T2-w, T2- weighted.

**Table 3:** Performance of different model.

Metrics	Clinical data-based model		Radiomics based model		Radiomics and clinical-radiomics based model	
	Training cohort	Validation cohort	Training cohort	Validation cohort	Training cohort	Validation cohort
C-Index	0.716	0.603	0.818	0.746	0.827	0.751
AUC	0.583	0.655	0.734	0.606	0.777	0.695
ACC	55.40%	67.40%	69.30%	64.00%	74.80%	69.80%
Sensitivity	43.30%	51.40%	63.30%	51.40%	82.50%	62.20%
Specificity	73.20%	79.60%	78.00%	73.50%	63.40%	75.50%

AUC, area under the ROC curve; ACC, accuracy.

### Validation of the Radiomic Nomogram

**Figure 2:**
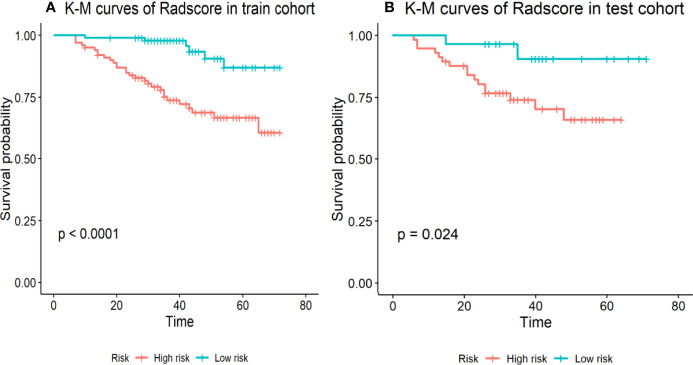
Stratified analyses were performed to estimate progression-free survival in the training cohort and the validation cohort; **(A)** K-M curves of Radscore in train cohort. **(B)** K-M curves of Radscore in test cohort. High-risk patients show a lower progression-free survival rate than low-risk patients, *p <*0.05.

In the training cohort, the concordance index, Sensitivity, and Specificity were 0.827 (95%CI: 0.757–0.898), 82.5%, and 63.4%, respectively, and in the validation cohort, they were 0.751 (95%CI: 0.641–0.861), 62.2%, and 75.5%, respectively. **Figures 1B, C** show the 1-, 3-, and 5-year survival calibration curves of the nomogram in training and validation cohorts, respectively. And there was good calibration in the two cohorts. Low-risk groups with higher radiomics signature had significantly better PFS than high-risk groups in both the training cohort and the validation cohort (*p* < 0.05; **Figure 2**).

## Discussion

Functional and anatomical MRI has been widely used to identify the therapeutic response in NPC (25). One study performed by Zhang et al. showed that the pretreatment ADC value was related to the neoadjuvant chemotherapy response for NPC patients (26), but its effectiveness is questioned. It has been indicated that MRI-based texture analysis demonstrated a good potential to predict the response to chemoradiotherapy of NPC patients (27). There is currently a lack of effective biomarkers for NPC response to IC. Our findings are concordant with those from a previous study that suggested that pretreatment MRI radiomics features could predict early response to IC in endemic NPC patients, but there was no additional validation cohort (28). In our research, we built a clinical radiomics nomogram to predict the response to IC pretreatment in NPC and classified these patients into high- and low-risk groups according to the median of the Radscores; the low-risk group had a significantly better PFS. It will help physicians tailor the treatment strategies and choose the most appropriate treatment. The radiomics signatures from the combination of T2-w and CET1-w images showed favorable predictive efficiency. The combined models established by the addition of MRI radiomics performed significantly better than that based on clinical data alone. One recent study showed a significant improvement in PFS prediction of NPC after incorporating the radiomics signature with the TNM system versus the TNM stage alone (15).

We chose two MRI sequences (T2-w and CET1-w) to extract image radiomics features, and 1,316 radiomics features were extracted for each NPC patient in total. Finally, the model was constructed based on 16 features consisting of 9 from enhanced T1-C and 7 from T2-w images. Our results demonstrated that features extracted from multivariate sequences performed significantly better than a single sequence. Image information from different sequences could more meaningfully reflect tumor heterogeneity and radiomics diversity.

To predict the prognosis of NPC, a number of studies on multivariate analysis of clinical factors were performed (29). Clinical factors include gender, age, TNM stage, lactate dehydrogenase (LDH), C-reactive protein, and plasma EBV-DNA levels. Some studies suggest that the pretreatment EBV DNA level could be used as an important biomarker for predicting the survival of NPC patients (30–32). It is similar to our findings: the pretreatment EBV DNA level was significantly associated with PFS. However, no significant association was observed between PFS with age or T-stage.

Many studies have demonstrated that radiomics features are related to tumor pathophysiologic features (33, 34). “Radio-genomics” is another emerging predicted tool (35) that combines radiomics with genomics data. Therefore, radio-genomics will be an attractive future direction in the prediction of NPC prognosis.

Our study had several limitations due to its retrospective nature. Firstly, because of the strict inclusion criteria, the sample size may not have been large enough to influence our results and final conclusion. Secondly, it was a single-center study, which may also limit the general applicability of our study results. We next aim to conduct multicenter prospective studies. Lastly, as a pathologic review of NPC patients would not be available, tumor response for IC assessed by MRI imaging based on anatomy might not be accurate. Furthermore, other MRI sequences, such as DWI, have been utilized to predict treatment response for breast cancer, and the potential value needs to be further explored in NPC (36).

In conclusion, our study developed a multiparametric MRI-based radiomics model to predict the NPC patient response to IC prior to treatment. It is helpful to choose NPC patients who will benefit from IC and then personalize their treatment (37).

## Data Availability Statement

The original contributions presented in the study are included in the article/**Supplementary Material**. Further inquiries can be directed to the corresponding author.

## Ethics Statement

This retrospective study was reviewed and approved by the institutional review Ethics Committee of Guangxi Medical University Cancer Hospital and was conducted in accordance with the Declaration of Helsinki. Due to the retrospective design of the study, the committee confirmed that the requirement for informed consent was waived.

## Author Contributions

GJ designed the study. LL, WP, HL, QW, DG, LJL, and DS performed study. LL, HL, and GJ wrote the manuscript. All authors read and approved the final manuscript. 

## Funding

This work was supported financially by the National Natural Science Foundation of China (Grant No. 81760533), the Natural Science Foundation of Guangxi Province(Grant No. 2018GXNSFAA281095). Guangxi Clinical Research Center for Medical Imaging Construction (Grant No. Guike AD20238096), Guangxi Key Clinical Specialty (Medical imaging Department), and Dominant Cultivation Discipline of Guangxi Medical University Cancer Hospital (Medical imaging Department. Guangxi: 139 program for Medical High-level Key Talents(Grant No. C202003014) and Youth Science Foundation of Guangxi Medical University(Grant No. GXMUYSF201925).

## Conflict of Interest

The authors declare that the research was conducted in the absence of any commercial or financial relationships that could be construed as a potential conflict of interest.

## Publisher’s Note

All claims expressed in this article are solely those of the authors and do not necessarily represent those of their affiliated organizations, or those of the publisher, the editors and the reviewers. Any product that may be evaluated in this article, or claim that may be made by its manufacturer, is not guaranteed or endorsed by the publisher.
