# Supplementary material for: A Clinical-Radiomics Nomogram Based on Magnetic Resonance Imaging for Predicting Progression-Free Survival After Induction Chemotherapy in Nasopharyngeal Carcinoma
Source: Front Oncol. 2022 Jun 22;12:792535. doi: 10.3389/fonc.2022.792535 (PMC9256909; doi:10.3389/fonc.2022.792535)
Supplement: Supplementary file 1 [file DataSheet_1.docx]

Supplementary Methods 1

LASSO in the “glmnet” package of R(version 3.6.2) was used to select radiomic features to fit the Cox proportion model. The Kaplan–Meier survival was analyzed using the “survminer” package running in R software. Cox proportional hazards regression analyses was performed with the “survival” package in R.Multi-factor nomogram and the calibration curveswere analyzed using the “rms” package running in R software. ROC curves and delong test were performed with the “pROC” package in R. The “reportROC” package running in R softwarewas used to calculate sensitivity and specificity. The “survcomp” package running in R softwarewas used to calculate cindex.
