# Supplementary material for: A Clinical-Radiomics Nomogram Based on Magnetic Resonance Imaging for Predicting Progression-Free Survival After Induction Chemotherapy in Nasopharyngeal Carcinoma
Source: Front Oncol. 2022 Jun 22;12:792535. doi: 10.3389/fonc.2022.792535 (PMC9256909; doi:10.3389/fonc.2022.792535)
Supplement: Supplementary file 2 [file DataSheet_2.docx]

Supplementary
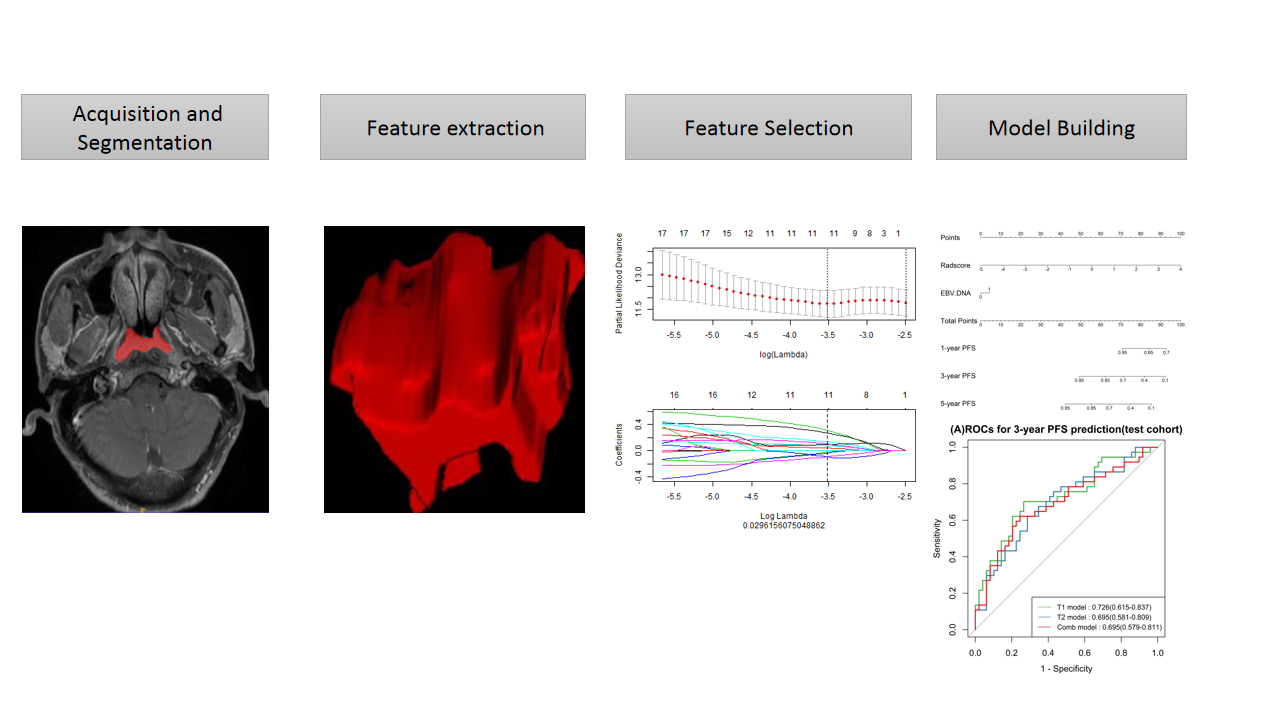
FIGURE 1 | Radiomics Workflow for predicting progression-free survival after Induction chemotherapy in Nasopharyngeal Carcinoma.
